# Involvement of Dynamic Adjustment of ABA, Proline and Sugar Levels in Rhizomes in Effective Acclimation of *Solidago gigantea* to Contrasting Weather and Soil Conditions in the Country of Invasion

**DOI:** 10.3390/ijms242015368

**Published:** 2023-10-19

**Authors:** Renata Bączek-Kwinta, Franciszek Janowiak, Magdalena Simlat, Jacek Antonkiewicz

**Affiliations:** 1 Department of Plant Breeding, Physiology and Seed Science, University of Agriculture in Krakow, ul. Podłużna 3, ul. Łobzowska 24, 30-239 Kraków, Poland; magdalena.simlat@urk.edu.pl; 2 The Franciszek Górski Institute of Plant Physiology, Polish Academy of Sciences, ul. Niezapominajek 21, 30-239 Kraków, Poland; f.janowiak@ifr-pan.edu.pl; 3 Department of Agricultural and Environmental Chemistry, University of Agriculture in Krakow, Al. Mickiewicza 21, 31-120 Kraków, Poland; jacek.antonkiewicz@urk.edu.pl

**Keywords:** abscisic acid, goldenrod, invasive plant species, osmotic adjustment, plant acclimation, random amplified polymorphic DNA

## Abstract

Giant goldenrod (*Solidago gigantea* Aiton) is one of the most invasive plant species occurring in Europe. Since little is known about the molecular mechanisms contributing to its invasiveness, we examined the natural dynamics of the content of rhizome compounds, which can be crucial for plant resistance and adaptation to environmental stress. We focused on rhizomes because they are the main vector of giant goldenrod dispersion in invaded lands. Water-soluble sugars, proline, and abscisic acid (ABA) were quantified in rhizomes, as well as ABA in the rhizosphere from three different but geographically close natural locations in Poland (50°04′11.3″ N, 19°50′40.2″ E) under extreme light, thermal, and soil conditions, in early spring, late summer, and late autumn. The genetic diversity of plants between locations was checked using the random amplified polymorphic DNA (RAPD) markers. Sugar and proline content was assayed spectrophotometrically, and abscisic acid (ABA) with the ELISA immunomethod. It can be assumed that the accumulation of sugars in giant goldenrod rhizomes facilitated the process of plant adaptation to adverse environmental conditions (high temperature and/or water scarcity) caused by extreme weather in summer and autumn. The same was true for high levels of proline and ABA in summer. On the other hand, the lowering of proline and ABA in autumn did not confirm the previous assumptions about their synthesis in rhizomes during the acquisition of frost resistance by giant goldenrod. However, in the location with intensive sunlight and most extreme soil conditions, a constant amount of ABA in rhizomes was noticed as well as its exudation into the rhizosphere. This research indicates that soluble sugars, proline, and ABA alterations in rhizomes can participate in the mechanism of acclimation of *S. gigantea* to specific soil and meteorological conditions in the country of invasion irrespective of plant genetic variation.

## 1. Introduction

Giant goldenrod (*Solidago gigantea* Aiton) is a perennial plant of the Asteraceae family and native to North America [1]. It is often mistakenly considered to be a harmless decorative plant, but both Europe and Asia have for years been under a threat of invasion by this species [1,2,3,4,5,6]. The risk of *Solidago* spread is increasing with the growth of global e-commerce [7]. Giant goldenrod is characterized by, among others, intensive growth, a large size, and a wide range of tolerance to adverse environmental conditions, as well as the production of allelopathic compounds [1,8,9]. It colonizes new areas through seeds, but after permanent takeover of a new area, its population grows quickly with the help of rhizomes, which also function as storage organs [1,3,8,9,10]. Fast growth and a large amount of rhizomes is the reason why *S. gigantea* decreases phosphorus content in the soil [11,12].

Water-soluble sugars are the main reserve material for plants, while changes in their concentration regulate metabolic and osmotic processes, gene expression, and consequently growth and development as well as stress signaling [13,14]. In the case of giant goldenrod, studies on the role of sugars in its metabolism have been conducted only on the above-ground plant part, in the context of the phytochemistry of *S. gigantea* as a medicinal plant [15,16].

Proline is a proteinogenic amino acid synthesized from glutamate in the cytoplasm and plastids. Upon environmental conditions triggering water scarcity in plants, it also serves as an osmoprotectant, cryoprotectant, signaling molecule, protein structure stabilizer, and ROS scavenger, allowing the plant to maintain a redox balance in adverse situations [17,18]. In the case of goldenrod, only the leaves of the other invasive goldenrod species, namely *S. canadensis*, were examined for proline changes in response to different light conditions [19]. However, its protective role for roots and rhizomes against osmotic stresses has been indicated for monocots [20,21,22] and the dicot *Glycyrrhiza glabra* [23]. Moreover, its function in root and shoot growth and development has been proven in different plant species [24,25,26].

As a “stress hormone”, abscisic acid (ABA) plays a crucial role in plant acclimation to various environmental stresses such as drought, cold, and heat stress, with substantial ABA accumulation usually observed under these conditions [27,28,29]. The response to water deficit in the form of stomata closure is the most characteristic role of ABA [30]. Additionally, during cold hardening, ABA levels increase significantly in the rhizomes of *Miscanthus × giganteus* plants [31]. The ABA-induced expression of plasma membrane aquaporins improves water flux through tissues [32]. Within cells, ABA induces the accumulation of stress protectants (small hydrophilic proteins, sugars, proline, and glycine betaine) and regulates the redox balance [33]. Moreover, slightly elevated ABA levels in roots stimulate their growth under water deficit [34,35].

RAPD markers are DNA fragments from polymerase chain reaction (PCR) where random segments of genomic DNA are amplified using a single primer with an arbitrary nucleotide sequence [36]. Despite various disadvantages (dominant inheritance and low reproducibility), RAPD markers are widely used for detecting polymorphism. Their usefulness, apart from purely technical aspects, results primarily from the possibility of generating a large number of markers that allow for a more general evaluation of the genome. RAPD markers were used, for example, to estimate the amount and distribution of genetic diversity within and among *Fritillaria tubiformis* subsp. *moggridgei* populations [37]. They were also used for the examination of the regional genetic structure in the invasive *Fallopia* complex occurring in different regions in Germany and Switzerland [38]. The genetic diversity existing in *Solidago canadensis* was also analyzed on the basis of RAPD marker distribution [39].

Thus, although the involvement of sugars, proline, and ABA in plant adaptation to environmental stress is well-documented, no studies have been conducted in this respect on the rhizomes of giant goldenrod. Meanwhile, by analyzing various adaptations, both developmental and physiological, a comprehensive assessment of the potential mechanisms of invasiveness can be carried out. Thus far, studies have been conducted on seed characteristics determining the invasiveness of giant goldenrod [2], but the ecological success of the introduced plants depends on many physiological factors [1,8]. Our study is the first attempt to estimate the dynamics of changes in three key groups of stress molecules, which can be crucial for the resistance of giant goldenrod to the drastically changing environmental conditions typical for the central and eastern European climate from late spring to late autumn, on soils of different origin and composition. We put forward the following hypotheses:

(1) Alterations in soluble sugars, proline, and abscisic acid contents in subsequent seasons of the year are associated with the *S. gigantea* response to changing environmental conditions typical for the vegetation period irrespective of plant genetic distance;
(2) The analyzed compounds reach their highest levels in late autumn, when the temperature drops, allowing plants to adapt to frost occurring in winter conditions;
(3) Soil conditions (mostly shallowness) affect the aforementioned responses.

## 2. Results

### 2.1. Soil Conditions in Individual Locations

The location numbered L1 was permanently shaded (Table 1) but had a comparable soil thickness to unshaded L3 (Table 2). The thickness of L2 (unshaded, Table 1) was only an approximately 30 cm-thin layer (Table 2) created from soil deposits and partially processed concrete. The pH was comparable in all stands (Table 2). The total nitrogen and phosphorus were lower in L3 than in L1 and L2 (Table 2). Nutrient and metal composition in L2 was the most extreme because of the highest levels of nutrients like organic C, Ca, Na, and S, and the highest levels of metals, namely Zn, Pb, and Al (Table 2).

On the basis of CF classification (Table 3), Ni and Al were ranked as low contamination (CF < 1) in all studied locations. Contrary, the CF of Cd indicated considerable contamination in all locations (3 < CF < 6). The differences between locations were related to Zn and Pb. In L2, stand Zn was ranked as considerable and Pb was moderate contamination, while in other stands, the CF values for Zn and Pb were ranked as low contamination.

### 2.2. Genetic Variation between Plants from Three Locations

**Figure 1:**
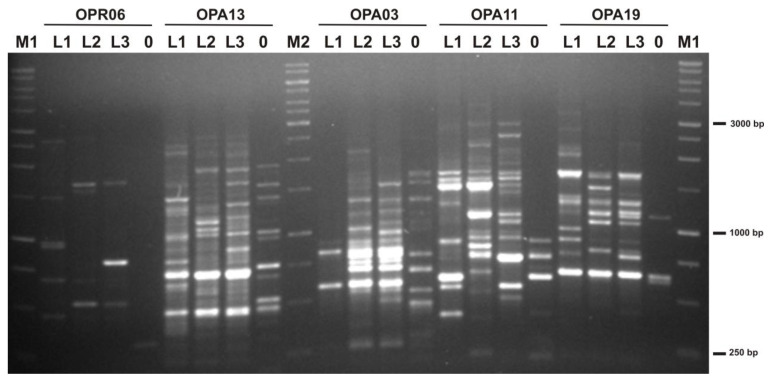
Gel electrophoresis of PCR products generated using RAPD primers OPR06, OPA13, OPA03, OPA11, and OPA19. L1, L2, L3—goldenrod; 0—negative control—sample without DNA; M1—1 kb DNA leader (EuRx; Gdańsk, Poland); M2—1 kb DNA leader (Thermo Scientific; Waltham, MA, USA).

All 15 decamer primers used in this study produced clearly identifiable bands that were used for further analysis (Figure 1). The range in size of the amplified products varied from about 250 bp to 4500 bp. The total number of the produced bands was 258, which averaged 17.2 bands per primer.

The number of polymorphic bands among the goldenrod locations (L1, L2, and L3) ranged from 7 for primer OPR-05 to 28 for primer OPB-07 (Table 4). On average, each primer produced 87.95 bands that showed polymorphism among plants taken from different locations.

Due to the very high proportion of polymorphic bands, similarity values among all analyzed locations were small and ranged from 0.2099 to 0.4326. The lowest similarity value was between goldenrods that were grown in L1 and L2 (0.2099), as well as the L1 and L3 (0.2278) locations. These values were about half as low compared to the value of the similarity index between the L2 and L3 goldenrods (0.4326; Table 5).

**Figure 2:**
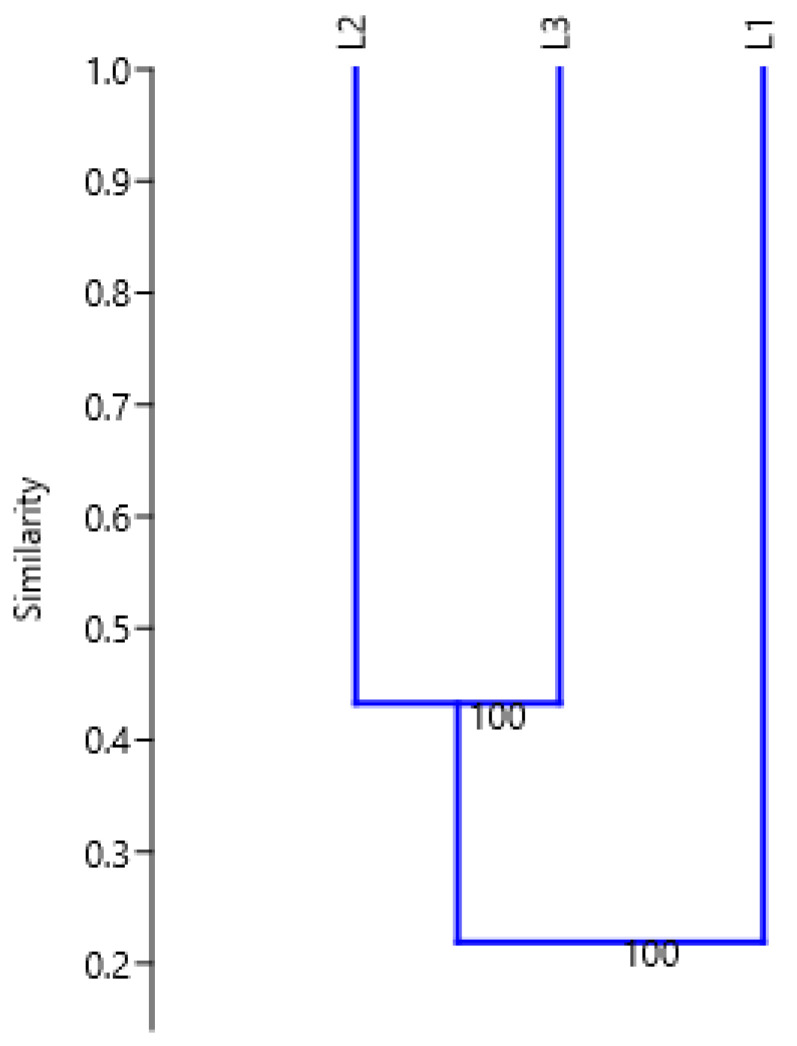
Dendrogram illustrating Jaccard’s similarity coefficient (cophenetic correlation r = 0.9974) for analyzed giant goldenrod accessions (L1, L2, and L3) by the UPGMA cluster analysis of the RAPD profiles derived using 15 primers.

As shown in the dendrogram (Figure 2) generated by cluster analysis using the UPGMA method based on Jaccard’s coefficient, L2 and L3 clustered together into one group whilst L1 was placed outside of this group.

### 2.3. In Vivo Measurements in Individual Stands

The greenness index obtained in spring was higher in L2 than in the other locations (Table 6). However, in summer, the values in this location dropped compared to spring, while they increased in L1 and L3. In autumn, leaves were dried so there was no point in performing the measurement.

In spring, plants were smaller in L1 than in the other locations (Table 6). In summer, the height of plants in L1 increased to 421% of the spring values, and in autumn, to 562%. In L3 in summer, the height increased by more than three times, and in autumn, by almost four times. Smaller values (314% and 322%) were obtained in L2, and in autumn, the shoots of these plants were the smallest.

### 2.4. Physiological Indicators in Rhizomes

The water content of goldenrod rhizomes was exerted mostly by the season (*p* = 0.000), then location (*p* = 0.002), and their interaction (*p* = 0.026), as the analysis of variance indicates (Table 7).

**Figure 3:**
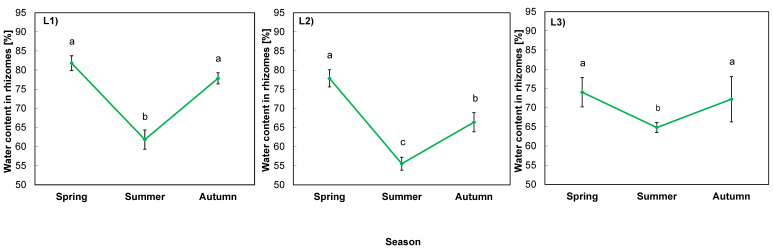
Water content in rhizomes of giant goldenrod grown in three different but geographically close natural locations (L1–L3). Plant growth conditions are shown in Table 1 and Table 2. Means from three biological replications ± SE are presented. Statistically different means are labeled with different letters (Duncan’s test; *p* ≤ 0.05).

The course of changes in water content is presented in Figure 3. In spring the values ranged from 74% (L3) to 82% (L1) of FW of rhizomes. In summer, the parameter dropped significantly to the level of ca. 60% in the rhizomes of plants in all locations. The largest decrease was observed in L2 with shallow soil, the highest content of toxic metals, and no shade. In autumn, there was an increase in all locations, but in L2, the water content was lower than in spring, while in the other locations it returned to the spring levels (Figure 3).

**Figure 4:**
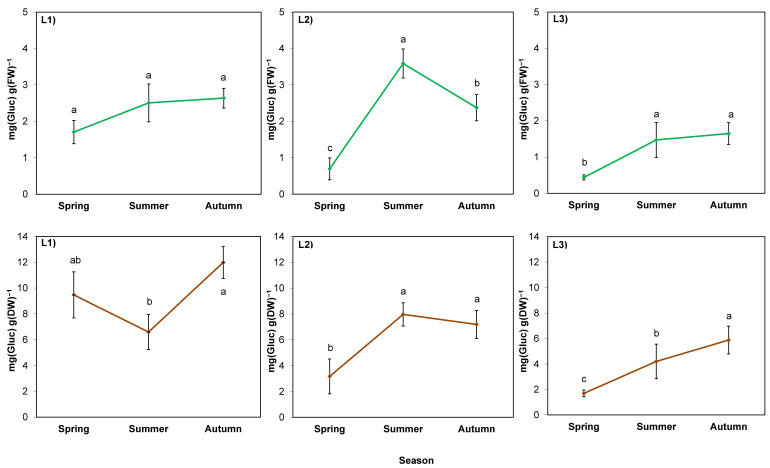
Water-soluble sugar content in rhizomes of giant goldenrod grown in three different but geographically close natural locations: L1–L3. Plant growth conditions are shown in Table 1 and Table 2. FW—fresh weight; DW—dry weight. Means from three biological replications ± SE are presented. Statistically different means are labeled with different letters (Duncan’s test; *p* ≤ 0.05).

In the case of soluble sugar content calculated per fresh weight (FW; Figure 4; green lines), the season was also a stronger factor than the location (*p* = 0.000 and 0.002, respectively), but there was no interaction of these factors (*p* > 0.05; Table 7). Sugar content increased in summer in L2 and L3, while L1 was characterized by high soil thickness and shade; the increase was insignificant. In L2, the increase was the largest (five-fold), followed by a decrease in autumn. In L3, the sugar content increased by three-fold and remained at the same level until autumn, similar to L1.

The course of the changes in sugar calculated per dry weight (DW; Figure 4; brown lines) of rhizomes in L2 and L3 (sunny) was similar to the course of the changes per FW. In contrast, in the rhizomes of plants in L1 (shaded), it was lower in summer than in autumn. Comparing the parameter between locations, it was the highest in L1.

**Figure 5:**
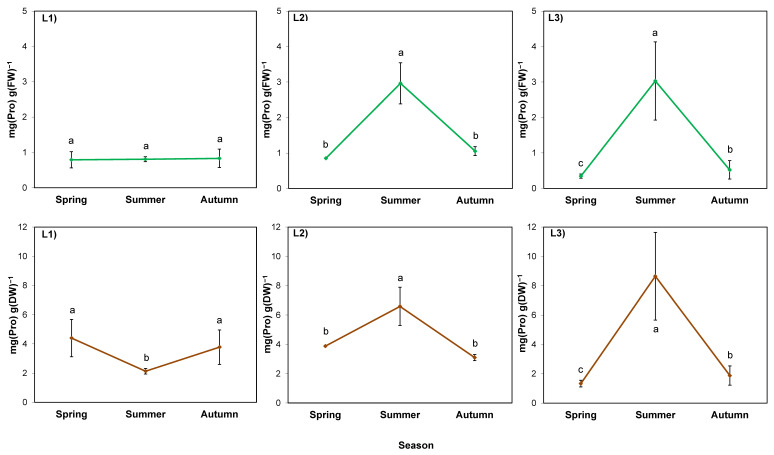
Proline content in rhizomes of giant goldenrod grown in three different but geographically close natural locations: L1–L3. Plant growth conditions are shown in Table 1 and Table 2. FW—fresh weight; DW—dry weight. Means from three biological replications ± SE are presented. Statistically different means are labeled with different letters (Duncan’s test; *p* ≤ 0.05).

The proline content calculated per FW of rhizomes was determined by the season (*p* = 0.008), but in a different manner in different locations (*p* = 0.029) (Table 7; Figure 5; green lines). This was manifested by an increase in proline content in summer in the sunny locations L2 and L3. It was also visible when calculated per DW of rhizomes from these locations (Figure 5; brown lines). Meanwhile, a decrease in proline content per DW was observed in summer in L1, similar to sugar content.

**Figure 6:**
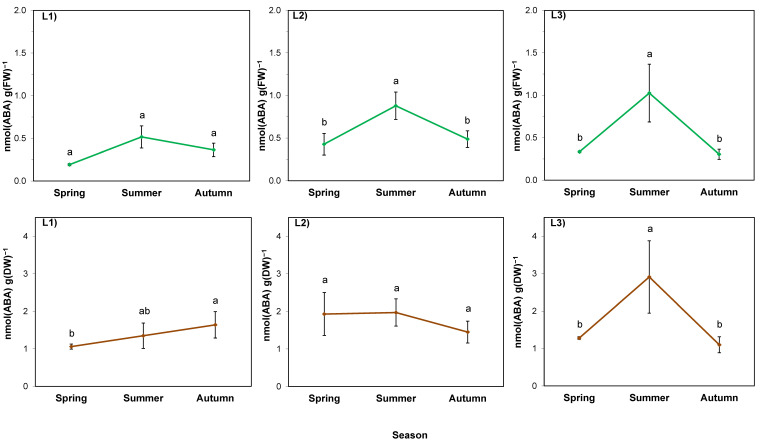
ABA content in rhizomes of giant goldenrod grown in three different but geographically close natural locations L1–L3. Plant growth conditions are shown in Table 1 and Table 2. FW—fresh weight; DW—dry weight. The background ABA content in the soil collected outside the goldenrod location was 0.037–0.072 nmol g^−1^ DW. Means from four biological replications ± SE are presented. Statistically different means are labeled with different letters (Duncan’s test; *p* ≤ 0.05).

The analysis of variance performed for abscisic acid per FW of rhizomes showed the effect of both factors as well as their interaction (Table 6; *p* < 0.05). The ABA level was primarily determined by the location (*p* = 0.000). It was higher in the rhizomes of plants growing in sunny locations (L2 and L3) than in the case of shaded L1, and on average was the highest in L2 throughout the entire studied period (Figure 6; green lines). In all locations, the ABA content increased in summer. In autumn, it returned to the spring levels in sunny locations, while in the shaded one it remained at the same level.

The average ABA level per DW in spring was similar in all locations (Figure 6; brown lines). In L1, the passage of seasons was accompanied by a continuous increase in its level, while in L2, it did not change throughout the entire studied period. In L3, on the other hand, there was an increase in summer followed by a decrease in autumn to the level obtained in spring, similar to the results calculated per FW.

The correlation between water content and ABA level was statistically significant, negative, and high (R > 0.7) irrespective of the location (Table 8). In the unshaded L2 stand with shallow soil, the correlation coefficient between water content and water-soluble sugar content in rhizomes was also statistically significant, negative, and high.

### 2.5. ABA in the Rhizosphere

**Figure 7:**
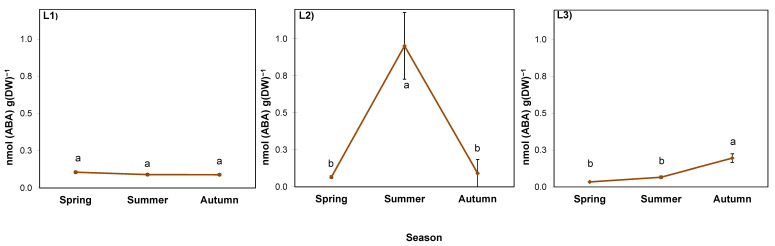
ABA content in the rhizosphere of giant goldenrod grown in three different but geographically close natural locations L1–L3. Plant growth conditions are shown in Table 1 and Table 2. DW—dry weight. The background ABA content in the soil collected outside the goldenrod locations was 0.037–0.072 nmol g^−1^ DW. Means from four biological replications ± SE are presented. Statistically different means are labeled with different letters (Duncan’s test; *p* ≤ 0.05).

The ABA level in goldenrod rhizosphere was determined by both experimental factors, as well as their interaction (Table 9). In the soil collected from a nearby cultivated field (considered background), it ranged from 0.037 to 0.072 nmol/g FW (mean 0.050 nmol/g FW; Figure 7). In shaded L1, it was twice as high as the soil background and constant at approximately 0.110 nmol/g FW. In L2, it was low in spring (0.063 nmol/g FW), but in summer it increased by several times to 0.953 nmol/g FW. Then, in autumn, it dropped back to the spring level. In L3, the highest ABA value, 0.195 nmol/g FW was noted in autumn (Figure 7).

## 3. Discussion

### 3.1. General Response of S. gigantea Plants in Studied Locations

At the moment, this is the first investigation of giant goldenrod rhizomes in regard to the response to changes in environmental conditions during the growing season in the country of invasion. The obtained data indicate that alterations in sugars, proline, and abscisic acid in subsequent seasons are associated with the plant response to changing meteorological conditions within the vegetation period. The response is also modified by the site specificity (shaded or sunny stand; differentiated soil conditions) in individual locations.

Interestingly, the most genetically distant plants in location L1 grew in the sole shaded location and in the thick soil. Therefore, genetic distance and soil thickness (shallowness) overlapped. However, soluble sugar, proline, and abscisic acid during the growing season differed between all locations, as well as between L2 and L3, where plants were genetically similar. Based on this, we conclude that the response of *S. gigantea* plants depends on changing environmental conditions in the specific habitat (location), not on genetic variation between *S. gigantea* plant clusters.

Czortek et al. [40] also concluded that both local soil properties and functional diversity affect the performance of *S. canadensis* in its invasive range in central Europe. On the other hand, Eckert et al. [41] pointed to some genetic, but not epigenetic adaptation processes of this species in its invasive range. Therefore, future studies on *Solidago* in central Europe should more strongly emphasize local and site-specific conditions and their impact on epigenetic variation. As Herrera et al. [42] reported, for *Helleborus foetidus*, that differences in local environmental features might play a similar or even greater role than spatial distance for epigenetic population structure, arguing for isolation by environment rather than isolation by distance.

It is noteworthy to reiterate that L2 and L3 were unshaded and daily exposed to solar irradiance, which is usually connected with increased air temperature [43]. The L2 edaphic conditions were the most extreme and it had the most contrasting composition, too, due to the highest level of nutrients, as well as the highest level of Al and heavy metals like Zn and Pb. Although Zn is a macronutrient, it can be toxic at high concentrations, while Pb and Al are unessential metals [44,45,46]. However, from the research of Dambiec et al. [47], *S. gigantea* can thrive in heavy-metal-polluted areas where Pb and Zn contents are much higher than in the soils analyzed in our research.

### 3.2. The Response of the Aerial Parts of Plants

The assessment of the condition of the above-ground plant parts was the reflection of plant condition, and for this reason it was considered the physiological background for the rhizome analyses in the subsequent seasons of the year. The greenness index and plant height data revealed that plants in L1 and L3, despite their genetic distance and different light conditions, invested in increasing chlorophyll content in summer. The reason was probably the high thickness of the soil allowing the roots to grow and perform their functions better than in L2 with its shallow soil and the highest amount of metals. It has to be mentioned that Al restricts root development while Zn and Pb affect chlorophyll formation [45,46], which was reflected in the lower grenness index in our study.

*S. gigantea* is considered to be a species that responds to changes in environmental conditions by adjusting its growth pattern, including shoot height [48], which was also noticed in our experiment. The smallest plant height observed in spring in L1 could be a result of delayed plant emergence in a shaded place with thick soil. In the sunny locations L2 and L3, the soil began to heat up earlier in the growing season, and *Solidago* rosettes could be noticed just in February/March. In summer (June–September), when the mean temperature in the studied area rose up to 20–25 °C, as seen in Figure 1, L1 plants could intensively perform life processes and produce biomass. At the same time, less access to light favors shoot elongation as a shade avoidance response, which is consistent with the study of Du et al. [19] on *S. canadensis* plants. In autumn, L1 and L3 plants with similar soil conditions reached the height typical for giant goldenrods—approximately 200 cm—and were larger than in L2. This could have resulted from the better accessibility of water and nutrients in the large volume of soil and the lack of a toxic effect of the heavy metals and Al on roots in the L1 and L3 soils. It is definitely not related to the genetic diversity of plants from individual locations, because according to the RAPD results, the higher similarity between plants from the L2 and L3 locations compared to L1 was detected.

### 3.3. Alterations in Water Content in Rhizomes

The underground parts of plants are not as prone to changing environmental conditions as the aerial parts [32,49,50]. In our experiment, water content changes in rhizomes were associated with soil properties and light conditions in the studied locations. The dynamics of these changes observed in L2 reflected the most stressful site to plants. The decrease in water content in summer occurred in all locations due to meteorological conditions, i.e., long period of high temperature and low precipitation. In autumn, the parameter returned to the values typical for rhizomes [49,51], but to a lesser extent in unshaded L2, with (already mentioned) the highest amounts of toxic metals in the soil, which could affect plant water management [44,45,46].

### 3.4. Accumulation of Sugars in Rhizomes can Be a Part of the Process of Giant Goldenrod Adaptation to Dry, Hot Summer and Pre-Winter Conditions

One of the hypotheses put forward in this work assumed that the sugar content in goldenrod rhizomes would increase during the hot, dry summer due to water stress. In L2, where the soil was shallow, and plants were directly exposed to sunlight and high temperature, which led to a sharp drop in the water content of rhizomes, there was an undeniable increase in the sugar content, regardless of the method of calculation. The opposite relationship between the two parameters was also confirmed by the correlation analysis. In the other locations, where the rhizomes grew in a thick soil layer conducive to water absorption and retention, changes in sugar content per FW were not as drastic, and the correlation coefficients between the water and sugar contents were not statistically significant. However, it is worth noting the difference between L1 (shaded) and L3 (unshaded), which indicates water stress, probably associated with the stronger evapotranspiration in L3. In L1, the sugar content in summer was close to the values obtained in spring, whereas in L3 there was an unquestionable increase, as in the case of L2. The synthesis and accumulation of water-soluble sugars is one of the strategies of plant adaptation to water scarcity caused directly (soil drought) or indirectly (high or low temperature and possibly other factors) [13,50,52].

The second hypothesis assumed that the increase in the studied compounds would occur in late autumn, because at that time the plants harden against frost, which in the central European climate can occur for shorter or longer periods from the beginning of November. In autumn of the studied year, the temperature dropped to −2.8 °C at the end of November, which combined with the shortened daylength, should trigger the hardening processes in plants, during which sugars are accumulated [53]. Sugars belong to the most important compounds enabling the frost resistance of plants by lowering the freezing point of cell sap (osmoregulation) [50]. Although the course of spring–autumn dynamics of sugars was dependent on the location and calculation method, compounds were definitely accumulated in autumn that prepared rhizomes for osmoregulation in cold conditions, protecting them against potential freezing.

In the case of L1, it is also worth considering the summer decrease in sugar content per DW in the context of anabolic processes allowing plants to accumulate biomass. To some extent, their determinants are large values of chlorophyll content (greenness index) and the rapid growth of the above-ground plant part—both discussed earlier. It can be supposed that plants in the L1 location invest in the growth of shoots, which can produce the next generative generation, seeds. In autumn, sugars become necessary for the survival of rhizomes providing vegetative reproduction in the following year, and therefore, they are accumulated again in the rhizomes of L1 plants.

### 3.5. Alterations of Proline and Abscisic Acid in Rhizomes Participate in Acclimation of S. gigantea to Local Drought and/or High-Temperature Stress, but Not to Pre-Winter Conditions

A summer increase in proline content in the open (unshaded) locations L2 and L3, as in the case of water-soluble sugars, can be a part of the osmoregulation necessary for plant adaptation and acclimation [18,25]. However, as there was no proline increment in autumn, and even a huge drop in the L2 and L3 locations, the hypothesis of the participation of proline in the winter hardening of *S. gigantea* failed. At the moment, there are no data on proline content in *Solidago* roots. It was analyzed only in leaves of *S. canadensis* in China, but in the context of shade response and plant plasticity [19].

The increase in the abscisic acid content of rhizomes observed in summer, regardless of the dynamics of particular locations, is another manifestation of *S. gigantea* acclimation to meteorological conditions in the studied season. Considering the pattern of changes for FW, it was similar in all stands, but the increase was noticed in sunny L2 and L3 locations. ABA accumulation enables plants to maintain root growth under moderate water stress [54], although there are no literature data about ABA levels in the rhizomes of giant goldenrod. Only one paper reported ABA levels (0.4–1.5 nmol/g FW) in the rhizomes of *S. canadensis* L. under different concentrations and nitrogen forms [55].

The correlation analysis, together with the analysis of Figs. 3 and 6, indicated that ABA changes were closely linked to water management in rhizomes in all locations, as any decrease in water content was accompanied by an increase in ABA levels, and conversely, when the water content increased, ABA levels decreased. This clearly indicates that ABA plays a key role in the adaptation of giant goldenrod plants to stressful growth conditions and is a part of the invasive mechanisms of this species.

It should also be mentioned that the mechanism of ABA action in rhizomes can be multidirectional, as this phytohormone is responsible for the expression of many genes involved in hardening and the resistance to various abiotic factors [56,57]. ABA is also conducive to the maintenance of stem cells in roots [58,59], and stimulates the susceptibility of roots to mycorrhiza and its functionality [60,61], which can lead to increased phosphorus uptake [11]. It can be extremely important for rhizome-creating plants like *Solidago*, because the phosphorus supply is important for the underground plant part growth and development [12]. Hence, further study on ABA changes in *Solidago* roots and rhizomes are worthy of being conducted.

As the emergence of new vegetative buds on rhizomes was observed, it cannot be ruled out that the high content of abscisic acid in rhizomes in summer, regardless of location and calculation method (FW/DW), can also be associated with the transition of the plant from the vegetative to the generative phase, which in goldenrod (short-day plant) occurs at the turn of August and September [62,63].

The presence of ABA in the rhizosphere of giant goldenrod and its increase is an intriguing phenomenon. As ABA inhibits the seed germination and growth of plants of other species [64,65], it might promote the invasiveness of *Solidago gigantea*. This phytohormone is often present in fallen leaves [66], but we did not observe any differences in plant litter between the studied locations, which means that ABA was definitely secreted by the goldenrod roots. According to Zhang et al. [67], root ABA stimulates rhizosheath formation by promoting root and root hair growth, so the larger soil layer adhering to the longer and denser roots allows plants to sustain high stomatal opening and photosynthesis during drought [68]. It occurs in environmental conditions similar to the ones in our study, namely soil drying and intermittent watering [67], which seemed especially important in the open and shallow L2 location in summer. The rhizosheath can also be important for *Solidago* in terms of increased P uptake [69], which could have occurred in the L3 location with the lowest P soil content among the studied stands.

To sum up, the present paper provides, for the first time, detailed time courses of changes in sugars, proline, ABA, and hydration levels in *Solidago gigantea* rhizomes in response to environmental stresses that the plants can experience in the country of invasion. Based on the meteorological data and soil and plant analyses obtained in the study, it can be stated that the adaptive potential to the environment of *S. gigantea* is broad, irrespective of its genetic diversity as indicated by RAPD. Soluble sugars, proline, and ABA alterations in rhizomes, as well as ABA secretion to the rhizosphere, can participate in the mechanism of acclimation of the most invasive plant species occurring in Europe with respect to dry and hot summer, soil shallowness, P deficiency, and decreased temperature in autumn.

## 4. Materials and Methods

### 4.1. Location and Plant Selection

**Figure 8:**
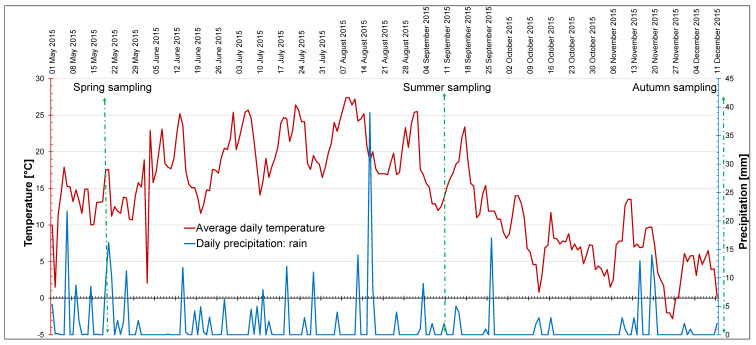
Average daily temperature and actual precipitation, starting from two weeks before the first sampling to the last day of sampling. Data from SatAgro (satagro.pl, 1 November 2020) for natural locations of giant goldenrod (50°04′11.3″ N, 19°50′40.2″ E). The measurement and sampling dates are marked.

The study was conducted in Kraków, Lesser Poland Province, southern Poland, central Europe. The area chosen for the study was temperate shrubland with anthropogenic paths of asphalt and concrete. Three locations with naturally growing plant clusters of giant goldenrod (*Solidago gigantea* Aiton) were selected, with different exposure to solar irradiation and soil conditions (Table 1 and Table 2 and Figure 8), but with a short distance between the sites (20–50 m; 50°04′11.3″ N, E 19°50′40.2″ E). The locations can be characterized as follows: L1 was permanently shaded and the soil was thick; L2 was open with shallow soil, but the richest in nutrients and heavy metals; and L3 was open with thick soil.

The choice of plants was performed based on (i) their morphology, important in species recognition (Botta-Dukát and Balogh 2008); and (ii) spatial distribution. As *Solidago* can reproduce both vegetatively and generatively and tiny local subpopulations can spread, molecular methods were also included to assess the genotypic variation of L1, L2, and L3 populations from the specific locations (see random amplified polymorphic DNA (RAPD) analysis).

### 4.2. Meteorological Data and Light Conditions In Situ

Daily temperature (mean) and daily precipitation data were obtained from SatAgro (satagro.pl). Figure 8 presents the data from 1 May 2015, i.e., 2 weeks before the first sampling, to 11 December 2015, when the last sampling took place. The vegetation season of 2015 was characterized by high mean temperature and low precipitation in general (Figure 8, Table 10). In the studied time period, the highest temperature was recorded on 8–9 August (27.4 °C), and the lowest daily temperature on 26 November (−2.8 °C). The mean air temperature over the studied period was 14 °C and the total precipitation was 345.1 mm (Table 10). The highest precipitation was recorded on 17 August (30 mm). On sampling days, there was no rainfall, or it was passing and minor—not exceeding 2 mm (Figure 8). Snowfall did not occur during the studied period. The seasons were defined on the basis of phytophenology of indicator plants typical for the local climate (www.agrometeo.pogodynka.pl/fenologia/fenologiczne_pory_roku, accessed on 1 November 2020). We referred to phenological stages, because in our opinion they characterize the seasons for vegetation better than meteorology, especially in the changing climate.

Total precipitation, mean precipitation, mean air temperature, and bioclimatic indices are indicated in Table 10. The De Martonne index (DMI) was calculated using the total precipitation over the studied period (P) and mean temperature (T_m_) for the studied period [70]. Ellenberg climate quotient (EQ) was calculated after [71] and [72] using the maximum temperature (T_max_) noticed in August and the total precipitation over the studied period. According to [70], DMI values between 15 and 24 indicate semi-arid conditions. An EQ equal to 30 is the border value between humid and dry climates, and levels above 30 characterize dryer and warmer regions [71,72]. A decreasing tendency in terms of rainfall quantity in the studied area was also emphasized by Jarosińska and Bodziony [73], who analyzed data from 18 precipitation-monitoring stations in Kraków.

Light conditions (Photosynthetic Photon Flux Density, PPFD) were assessed on the day of sampling at the plant level (position of leaves 1–5, counting from the top) using QSPAR Quantum Sensor (Hansatech Instruments, Kings Lynn, UK; Table 1).

### 4.3. Plant Analyses In Situ and Plant Material Collection

**Figure 9:**
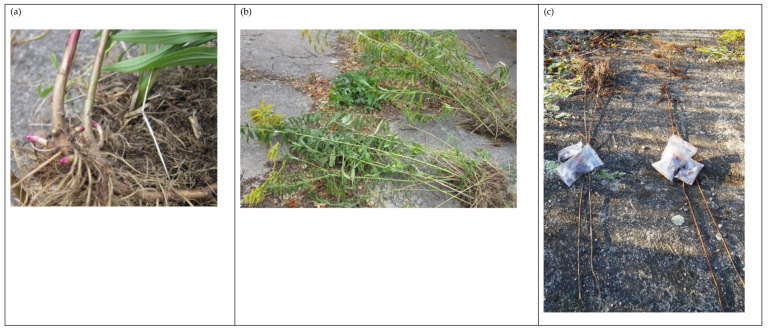
Giant goldenrod rhizomes in spring (**a**); full-grown blooming plants in summer (**b**); and desiccated plants in autumn (**c**). Example of plants from L3 stand.

Plants used for measurements and taken for analysis were selected from the central part of the L1–L3 clusters of at least 1 m^2^, surrounded by other goldenrod plants, so the microclimate specific to each location was preserved. The experimental areas were marked with wooden stakes. Sampling was performed in spring (15 May), summer (11 September), and autumn (11 December) (see Figure 8) because in the climate of central Europe, the weather conditions always drastically differ in those seasons. Before sampling, the basic indicators of plant condition were checked, namely each plant’s height and leaf greenness index. The latter was determined (provided that the leaves were viable) using the CL-01 m (Hansatech Instruments, Kings Lynn, UK; Table 3). The greenness index reflects the content of chlorophyll (Chl) *a* and *b* [74]. Chl *a* and *b* absorb red light but do not absorb infrared light. The CL-01 m measures the absorbance in defined areas, yielding numerical values proportional to Chl content, as was used in our previous studies on different plant species [75,76]. The measurement was performed on leaves between the 3rd and 5th node counting from the top of the plant. Basic photographic documentation was also assembled (Figure 9).

For RAPD analysis, the leaf material from 10 randomly selected plants per one location (L1, L2, and L3) was immediately frozen in string plastic bags in liquid nitrogen and stored in a deep freezer (−70 °C) before DNA extraction.

Rhizomes (basal and distal parts up to 20 cm in length) of the same plants were carefully extracted together with the bulk soil containing rhizomes. This was performed once per season from each location (three plants per location; see Figure 8 for the sampling dates). The collected material was placed in string plastic bags and immediately stored in a deep freezer at the temperature of ca. −70 °C. The frozen material was quickly and gently cleaned of soil with a soft brush and weighed, and then divided into fragments of approximately 100 to 500 mg for further analysis.

### 4.4. Soil Collection and Analyses

Soil thickness (the depth from the topsoil profile to the weathered bedrock) was assayed after digging into the soil profile. Soil samples taken from the depth of 0–30 cm were pooled within the location, then the soil material was dried in a forced-air circulation dryer at 70 °C. After drying and sifting through a 2 mm sieve, soil pH was determined by a potentiometric method in 1 mol ∙ dm^−3^ KCl. Organic carbon was evaluated by the Tiurin method, and total nitrogen content by the Kjeldahl method. The content of available P was established using the Egner–Riehm method [77,78].

The levels of macronutrients Ca, Na, and S and metals Zn, Ni, Cd, Pb, and Al were assessed after digesting the soil in a mixture of concentrated acids: HNO_3_ (65%) and HClO_4_ (70%) (3:2, *v*/*v*). Then, the assays with atomic emission spectrometer Optima 7300 DV Spectrometer ICP-OES (Perkin Elmer, Waltham, MA, USA) were performed [77,78]. Each sample was analyzed in three replicates and data were analyzed using a quantitative analysis mode. Scanning of each sample was also repeated three times to gather repetitive results. During the measurements, a wash-out time of 0.5 min was used to avoid the memory effect [79]. Accuracy of the analytical methods was verified based on certified reference materials: CRM IAEA/V—10 Hay (International Atomic Energy Agency), CRM—CD281—Rey Grass (Institute for Reference Materials and Measurements), CRM023-050—Trace Metals—Sandy Loam 7 (RT Corporation).

The contamination factor was calculated as the ratio of the concentration of each metal assayed in the soil collected from each location and the concentration of the respective metal in the background. It was calculated according to the following equation:

CF = C_n_/B_n_

where C_n_ is the concentration of the metal found in the soils and B_n_ is the background value of the metal [80]. If the CF is described as < 1, then it means “low contamination” 1 < CF < 3 = “moderate contamination” 3 < CF < 6 = “considerable contamination” and CF > 6 = “very high contamination” [81]. According to [82], we considered the values from the upper continental crust (UCC) as the background (B_n_). The background values of Zn, Ni, Cd, Pb, and Al in the UCC are 52 μg/g, 18.6 μg/g, 0.102 μg/g, 17 μg/g, and 77440 μg/g, respectively.

### 4.5. Random Amplified Polymorphic DNA (RAPD) Analysis on Leaves

DNA was isolated from bulk samples of leaves from each location. The samples were ground in liquid nitrogen using mortar and pestle, and 100 mg of the obtained tissue powder was transferred into 1.5 mL Eppendorf tube. For DNA isolation the Genomic Mini AX Plant Kit (AA Biotechnology; Gdańsk, Poland) was used following the manufacturer’s instruction. Quality and quantity of DNA were monitored by both gel electrophoresis (1% agar gel in 1xTBE buffer) and spectrometric measurements (NanoDrop 2000c; Thermo Scientific, Waltham, MA, USA). PCR was performed in a total volume of 20 μL containing 20 ng of template DNA. Fifteen RAPD primers were used, and the reaction mixture was prepared according to Simlat et al. [83]. The amplifications were carried in Eppendorf Mastercycler Gradient, and PCR products were resolved in 1.2% agarose gel containing ethidium bromide at a concentration of 0.4 μg/mL. Gels were run in 1xTBE buffer at 6 V/cm for 3 h and examined in UV light. Amplification with each primer was performed in duplicate to control band regularity, and only the bands present or absent in both replications were used for consideration. The presence or absence of an individual band was scored as 1 or 0, respectively, and polymorphism analysis was performed according to Simlat et al. [83].

### 4.6. Measurement of Rhizome Water Content

Water content and dry weight (DW) were estimated for the rhizomes (n = 3) with a balance (AS 220.R2, Radwag, Radom, Poland, d = 0.001), before and after drying of the rhizome samples with fresh weight (FW) of approx. 500 mg at 100 °C for 24 h.

### 4.7. Measurement of Rhizome Sugar Content

Sugars were assayed spectrophotometrically with the anthrone method [84]. Rhizome samples (n = 3) with FW of 250 mg were homogenized in a mortar in 10 mL of distilled water, then heated for 15 min in a water bath at 90 °C and centrifuged for 10 min at 5000 rpm (5430R; Eppendorf, Hamburg, Germany). Anthrone reagent (2 mL; 1 mg of anthrone; Sigma-Aldrich, Darmstadt, Germany) per 100 mL of concentrated H_2_SO_4_, analytical grade, (ChemPur, Piekary Śląskie, Poland) was added to a cooled and 10-fold-diluted sample. The absorbance of the complex compound was measured at the wavelength of 620 nm (Ultrospec 2100; Amersham, UK). The results were referenced to the calibration curve obtained for glucose (Sigma-Aldrich) at the concentration 0.0039–0.0312 mg per 1 mL. The control was a sample of 1 mL distilled water.

### 4.8. Measurement of Rhizome Proline Content

Proline was determined spectrophotometrically with the ninhydrin method based on [85,86] and [87]. Rhizomes (3 portions of approx. 100 mg FW) were homogenized with 2 mL of an ethanol:water mixture (70:30, *v*/*v*). The homogenates were centrifuged for 5 min at 16,000× *g* (Minispin; Eppendorf, Hamburg, Germany). Ninhydrin (1%; Sigma-Aldrich, Darmstadt, Germany) in a mixture of acetic acid (analytical grade, ChemPur, Piekary Śląskie, Poland, 60%, *v*/*v*) and ethanol (analytical grade, ChemPur, Poland, 20%, *v*/*v*) was added to the supernatant and heated in a water bath at 90 °C for 20 min in darkness. After cooling at room temperature in darkness, the samples were centrifuged again (1 min, 16,000× *g*), transferred to another set of tubes, and the absorbance was read at the wavelength of 520 nm (Ultrospec 2100; Amersham, UK). The calibration curve was prepared for 0.0625–1 mM proline (Sigma-Aldrich) in an ethanol:water mixture (70:30, *v*/*v*).

### 4.9. Measurement of Abscisic Acid Content in the Rhizomes and Rhizosphere

Rhizomes (n = 3) were freeze-dried and samples (30–150 mg DW) were ground (6 min, 25 cycles/s) with a ball mill MM400 (Retsch, Haan, Germany) in Eppendorf vials. After adding 1.5 mL of cold, redistilled water, the vials were heated for 3 min in a thermoblock set to 90 °C and then shaken overnight (approx. 18 h) using Yellow Line OS 5 Basic at 560 cycles/min at 4 °C in CH500 Angelantoni chamber in order to extract ABA. The next day, the aqueous extracts were centrifuged for 20 min in a refrigerated centrifuge (MPW-350R, Warsaw, Poland) at 18,000× *g*. ABA was measured in the supernatant using indirect enzyme-linked immunosorbent assay (ELISA) according to Walker-Simmons and Abrams [88]. The antibody used was MAC 252 (Babraham Technix, Cambridge, UK). Absorbance was measured with a microplate reader Model 680 (Bio-Rad Laboratories, Hercules, CA, USA) at the wavelength of 405 nm. For each treatment, at least eight ELISA measurements were performed on four independent biological samples.

Three soil samples of the rhizosphere and bulk soil collected outside the goldenrod locations (ca. 3 mL) for each location and sampling date were collected, freeze-dried, and then weighed (DW). After adding 3 mL of cold, redistilled water, the samples were shaken overnight and then centrifuged, after which ABA was measured in the supernatant—as described for rhizomes.

### 4.10. Statistical Analysis

Non-destructive analyses (greenness index; plant height) in each location were performed in three biological replications for each season, on the same plants. The greenness index was measured for the leaves of three plants, between the 3rd and 5th nodes counting from the top of the plant. Each leaf was analyzed three times. Rhizome analyses from the same plants were performed in three to four biological replications (rhizome cuts), each in at least three instrumental replications. The results were processed using Microsoft Excel and STATISTICA 10.0 (Statsoft Inc.). First, normality distribution was checked with the Shapiro–Wilk test. Then, to determine the significance of the differences within the sampling date (season) or location, Duncan’s test at *p* ≤ 0.05 was used for three means and Student’s *t*-test at *p* ≤ 0.05 for two means. In the case of water content and the level of metabolites calculated per FW, the results of the variance analysis in a two-factorial design are also provided. The first factor was the season in which the measurement was performed and/or the sample was collected, and the second factor was the location. Correlation coefficients between physiological indicators in rhizomes were also calculated.

## 5. Conclusions

1. The response of *S. gigantea* plants depends on changing environmental conditions in the specific habitat (location), not on genetic distance, because soluble sugar, proline, and abscisic acid in rhizomes during the growing season differ between all locations, as well as between L2 and L3, which are genetically similar.
2. In all locations, the soluble sugar accumulation in goldenrod rhizomes is high in pre-winter conditions, which is definitely a part of winter hardening.
3. Soil shallowness triggers intensive metabolic changes in goldenrod plants towards the accumulation of sugars and ABA in L2 rhizomes.
4. Giant goldenrod plants grown in shaded locations invest their resources into shoot growth in summer to obtain more light, and during the vegetative season the changes in soluble sugar, proline, and abscisic acid in their rhizomes are moderate. Meanwhile, plants growing in the two unshaded locations, thus being more exposed to drought and high-temperature stress, inhibit anabolic processes in summer, which is reflected in the low chlorophyll content and shorter the aerial plant part.
5. During the growing season, changes in the content of sugars and proline in rhizomes are greater than the changes in ABA level, which indicates their significant participation in plant metabolism and response to meteorological and soil factors. At the same time, in sun-exposed locations, ABA content in rhizomes and the rhizosphere is high, which suggests its role in the survival of giant goldenrod rhizomes in sites prone to continuous solar irradiation combined with high temperature, water scarcity, toxic metals, and P deficiency.

## Figures and Tables

**Table 1:** Light conditions of three natural locations of giant goldenrod used in the study. The measurement was performed on the day of plant sampling (see Materials and Methods). The distances between the locations were 20–50 m.

Location Number	Description	PPFD (µmol (Quantum) m^−2^ s^−1^)		
		Spring	Summer	Autumn
L1	Permanent shade caused by the proximity of tall trees	42–406	1134–652	32–212
L2	No shade; plants lit with natural sunlight for the most part of the day	803–1198	830–1299	153–469
L3	No shade; plants lit with natural sunlight for the most part of the day	807–1122	817–1301	149–484

PPFD—photosynthetic photon flux density.

**Table 2:** Soil parameters of three natural locations of giant goldenrod used in the study. The distances between the locations were 20–50 m. The range (for thickness) or means ± SE (for other parameters) of three replicates are presented. DW—dry mass.

Parameter	Unit	L1	L2	L3
Thickness	cm	100–204 ^a^	5–32 ^c^	53–107 ^b^
pH_(H2O)_	-	5.82 ± 0.003 ^a^	5.71 ± 0.006 ^a^	5.73 ± 0.003 ^a^
pH_(KCl)_	-	5.66 ± 0.013 ^a^	5.32 ± 0.001 ^a^	5.35 ± 0.024 ^a^
Macronutrients				
C organic	%	1.948 ± 0.003 ^b^	2.355 ± 0.013 ^a^	1.885 ± 0.016 ^c^
N total	%	0.204 ± 0.001 ^a^	0.209 ± 0.001 ^a^	0.186 ± 0.002 ^b^
P	mg ∙ kg^−1^ DW	118.7 ± 1.4 ^a^	92.4 ± 1.5 ^b^	53.3 ± 0.1 ^c^
Ca	mg ∙ kg^−1^ DW	2395.6 ± 23.7 ^b^	3015.9 ± 22.3 ^a^	2348.7 ± 24.9 ^b^
Na	mg ∙ kg^−1^ DW	4.52 ± 0.38 ^b^	10.48 ± 0.87 ^a^	9.09 ± 1.04 ^a^
S	mg ∙ kg^−1^ DW	25.7 ± 0.7 ^b^	38.7 ± 0.6 ^a^	26.5 ± 0.4 ^b^
Metals				
Zn	mg ∙ kg^−1^ DW	43.1 ± 1.4 ^b^	212.3 ± 2.8 ^a^	44.2 ± 0.4 ^b^
Ni	mg ∙ kg^−1^ DW	1.11 ± 0.03 ^a^	0.86 ± 0.01 ^b^	1.21 ± 0.01 ^a^
Cd	mg ∙ kg^−1^ DW	0.60 ± 0.01 ^a^	0.33 ± 0.00 ^b^	0.35 ± 0.00 ^b^
Pb	mg ∙ kg^−1^ DW	10.8 ± 0.0 ^c^	23.3 ± 0.1 ^a^	15.1 ± 0.2 ^b^
Al	mg ∙ kg^−1^ DW	530.7 ± 21.7 ^b^	619.4 ± 4.6 ^a^	530.8 ± 5.6 ^b^

Statistically different means are labeled with different letters (Duncan’s test; *p* ≤ 0.05).

**Table 3:** Contamination factor values for metals assayed in the soils from three natural locations of giant goldenrod used in the study. The distances between the locations were 20–50 m.

Metal	Location		
	L1	L2	L3
Zn	0.83	4.08	0.85
Ni	0.06	0.05	0.07
Cd	5.88	3.24	3.43
Pb	0.64	1.37	0.89
Al	0.01	0.01	0.01

**Table 4:** The number of amplified giant goldenrod DNA fragments via RAPD primers.

Primer	Primer Sequence (5′→3′)	Total Number of Amplified Bands	The Number of Polymorphic Bands	% of Polymorphic Bands
OPA-01	CAGGCCCTTC	20	16	80.0
OPA-02	TGCCGAGCTG	13	9	69.2
OPA-03	AGTCAGCCAC	18	14	77.8
OPA-10	GTGATCGCAG	13	13	100.0
OPA-11	CAATCGCCGT	17	15	88.2
OPA-13	CAGCACCCAC	20	16	80.0
OPA-19	CAAACGTCGG	15	15	100.0
OPB-07	GGTGACGCAG	30	28	93.3
OPB-11	GTAGACCCGT	16	16	100.0
OPR-01	TGCGGGTCCT	15	9	60.0
OPR-02	CACAGCTGCC	23	19	82.6
OPR-03	ACACAGAGGG	14	14	100.0
OPR-04	CCCGTAGCAC	20	20	100.0
OPR-05	GACCTAGTGG	7	7	100.0
OPR-06	GTCTACGGCA	17	15	88.2
Total		258	226	
Average/primer		17.20	15.13	87.95

**Table 5:** Similarity matrix of analyzed giant goldenrod accessions (L1, L2, and L3) generated on the basis of RAPD data using Jaccard’s similarity coefficient.

	L1	L2	L3
L1	1.0000		
L2	0.2099	1.0000	
L3	0.2278	0.4326	1.0000

**Table 6:** Basic indicators of plant condition: greenness index of leaves at the 3rd to 5th node counting from the top, and the height of giant goldenrod plants in different seasons in three natural locations. Meteorological conditions are given in Material and Methods. Light conditions on the day of the measurements and soil conditions are presented in Table 1 and Table 2, respectively.

Location	Greenness Index (CL-01, Arbitrary Units)			Plant Height (cm)		
	Season			Season		
	Spring	Summer	Autumn	Spring	Summer	Autumn
L1	8.68 ^b,B^ 100%	13.1 ^a,A^ 151%	Leaves dried	37.3 ^c,B^ 100%	157.3 ^b,B^ 421%	209.7 ^a,A^ 562%
L2	10.9 ^a,A^ 100%	8.59 ^b,B^ 79%	Leaves dried	53.2 ^b,A^ 100%	166.7 ^a,AB^ 314%	171.3 ^a,B^ 322%
L3	7.90 ^b,B^ 100%	13.5 ^a,A^ 171%	Leaves dried	52.7 ^c,A^ 100%	179.0 ^b,A^ 340%	206.3 ^a,A^ 391%

Means from three biological replications are provided together with the percentage of values obtained in spring. Significance of differences was analyzed within location (lower-case letters) and season (upper-case letters). Different letters (A vs. B or a vs. b, etc.) indicate that the data differ. In the case of greenness index, Student’s *t*-test at *p* ≤ 0.05 was used, and in the case of plant height, Duncan’s test at *p* ≤ 0.05.

**Table 7:** Variance analysis of water, sugar, proline, and ABA content per fresh weight (FW) of giant goldenrod rhizomes in different seasons and in three different natural locations. Plant growth conditions are presented in Table 1 and Table 2.

Factor	Water Content		Sugars		Proline		ABA	
	F	*p*	F	*p*	F	*p*	F	*p*
Season (S)	53.10	0.000 ***	16.36	0.000 ***	13.26	0.008 **	8.51	0.002 **
Location (L)	8.79	0.002 **	8.92	0.002 **	2.82	0.086	33.83	0.000 ***
S × L	3.55	0.026 *	2.69	0.065	3.46	0.029 *	3.39	0.027 *

The significance of the effect of a given factor is marked: * *p* ≤ 0.05; ** *p* ≤ 0.01; *** *p* ≤ 0.001.

**Table 8:** Correlation coefficients between analyzed metabolites in giant goldenrod rhizomes in three natural locations, based on data related to FW.

Parameters Tested for Correlation	Correlation Coefficient in Specific Location		
	L1	L2	L3
Water content/water-soluble sugar content	−0.4386 ns	−0.8103 **	−0.5368 ns
Water content/proline content	0.0191 ns	−0.3183 ns	−0.5447 ns
Water content/ABA content	−0.7489 **	−0.7580 **	−0.7156 **
Water-soluble sugar content/proline content	−0.2260 ns	0.5357 ns	0.0764 ns
Water-soluble sugar content/ABA content	0.6286 ns	0.5750 ns	0.3024 ns
ABA content/proline content	0.0931 ns	0.1757 ns	0.5335 ns

**—statistically significant at *p* ≤ 0.01; ns— not statistically significant.

**Table 9:** Variance analysis of water and ABA content in the rhizosphere around giant goldenrod rhizomes in different seasons and in three different natural locations. Plant growth conditions are presented in Table 1 and Table 2.

Factor	ABA	
	Test Value F	*p*
Season (S)	326.9	0.002 **
Location (L)	294.3	0.000 ***
S × L	341.5	0.000 ***

The significance of the effect of a given factor is marked: ** *p* ≤ 0.01, *** *p* ≤ 0.001.

**Table 10:** Climatic and bioclimatic indices within the studied area.

Parameter	Description/Formula and Unit	Value
P *	Total precipitation [mm]	345.1
P_m_ *	Mean precipitation [mm]	1.53
T_m_ *	Mean air temperature [°C]	14.0
T_max_ *	Maximal air temperature [°C]	27.4
De Martonne aridity index (DMI) *	P/(T_m_ + 10) [mm/°C]	14.4
Ellenberg climate quotient (EQ) *	(T_max_ * × 1000)/P	31.5

* Over the studied period.

## Data Availability

The data presented in this study are available on request from the corresponding author. The data are not publicly available due to their planned reuse in a thesis.

## Abbreviations

MDPI	Multidisciplinary Digital Publishing Institute
DOAJ	Directory of open access journals

## References

[1] Weber E., Jakobs G. (2005). Biological flora of central Europe: *Solidago gigantea* Aiton. Flora.

[2] Bochenek A., Synowiec A., Kondrat B., Szymczak M., Lahuta L.B., Gołaszewski J. (2016). Do the seeds of *Solidago gigantea* Aiton have physiological determinants of invasiveness?. Acta Physiol. Plant.

[3] Botta-Dukát Z., Balogh L. (2008). The Most Important Invasive Plants in Hungary.

[4] Guo S., Fang F. (2003). Physiological adaptation of the invasive plant *Solidago canadensis* to environments. Acta Phytoecol. Sin..

[5] Tokarska-Guzik B., Bzdęga K., Nowak T., Urbisz A., Węgrzynek B., Dajdok Z. (2015). Propozycja Listy Roślin Gatunków Obcych, Które Mogą Stanowić Zagrożenie dla Przyrody Polski i Unii Europejskiej.

[6] Yuan Y., Wang B., Zhang S., Tang J., Tu C., Hu S., Yong J.W.H., Chen X. (2013). Enhanced allelopathy and competitive ability of invasive plant *Solidago canadensis* in its introduced range. J. Plant Ecol..

[7] Lenda M., Skórka P., Kuszewska K., Moroń D., Bełcik M., Bączek-Kwinta R., Janowiak F., Duncan D.H., Vesk P.A., Possingham H.P. (2021). Misinformation, internet honey trading and beekeepers drive a plant invasion. Ecol. Lett..

[8] Meyer G.A., Hull-Sanders H.M. (2008). Altered patterns of growth, physiology and reproduction in invasive genotypes of *Solidago gigantea* (*Asteraceae*). Biol. Invasions.

[9] Szymura M., Szymura T.H. (2015). Growth, phenology, and biomass allocation of alien *Solidago* species in central Europe. Plant Species Biol..

[10] Liao H., Gurgel P.C.S., Pal R.W., Hooper D., Callaway R.M. (2016). *Solidago gigantea* plants from nonnative ranges compensate more in response to damage than plants from the native range. Ecology.

[11] Majewska M.L., Rola K., Zubek S. (2017). The growth and phosphorus acquisition of invasive plants *Rudbeckia laciniata* and *Solidago gigantea* are enhanced by arbuscular mycorrhizal fungi. Mycorrhiza.

[12] Stefanowicz A.M., Stanek M., Nobis M., Zubek S. (2017). Few effects of invasive plants *Reynoutria japonica*, *Rudbeckia laciniata* and *Solidago gigantea* on soil physical and chemical properties. Sci. Total Environ..

[13] Hanson J., Smeekens S. (2009). Sugar perception and signaling—An update. Curr. Opin. Plant Biol..

[14] Hey S.J., Byrne E., Halford N.G. (2010). The interface between metabolic and stress signalling. Ann. Bot..

[15] Lendl A., Reznicek G. (2007). Two new saponins from *Solidago gigantea*. Sci. Pharm..

[16] Ratiu I.A., Al-Suod H., Ligor M., Ligor T., Railean-Plugaru V., Buszewski B. (2018). Complex investigation of extraction techniques applied for cyclitols and sugars isolation from different species of *Solidago genus*. Electrophoresis.

[17] Verbruggen N., Hermans C. (2008). Proline accumulation in plants: A review. Amino Acids.

[18] Verslues P.E., Sharma S. (2010). Proline metabolism and its implications for plant-environment interaction. Arab. Book.

[19] Du L.S., Liu H.Y., Yan M., Li J.M., Li J.S. (2017). Individual plasticity of the shade response of the invasive *Solidago canadensis* in China. PLoS ONE.

[20] Guan C., Cui X., Liu H.Y., Li X., Li M.Q., Zhang Y.W. (2020). Proline biosynthesis enzyme genes confer salt tolerance to switchgrass (*Panicum virgatum* L.) in cooperation with polyamines metabolism. Front. Plant Sci..

[21] Xie E., Wei X.J., Ding A.Z., Zheng L., Wu X.N., Anderson B. (2020). Short-term effects of salt stress on the amino acids of *Phragmites australis* root exudates in constructed wetlands. Water.

[22] Xiong J., Zhang L., Fu G., Yang Y., Zhu C., Tao L. (2012). Drought-induced proline accumulation is uninvolved with increased nitric oxide, which alleviates drought stress by decreasing transpiration in rice. J. Plant Res..

[23] Behdad A., Mohsenzadeh S., Azizi M., Moshtaghi N. (2020). Salinity effects on physiological and phytochemical characteristics and gene expression of two *Glycyrrhiza glabra* L. populations. Phytochemistry.

[24] Biancucci M., Mattioli R., Moubayidin L., Sabatini S., Costantino P., Trovato M. (2015). Proline affects the size of the root meristematic zone in *Arabidopsis*. BMC Plant Biol..

[25] Lehmann S., Funck D., Szabados L., Rentsch D. (2010). Proline metabolism and transport in plant development. Amino Acids.

[26] Mattioli R., Costantino P., Trovato M. (2009). Proline accumulation in plants. Plant Signal. Behav..

[27] Janowiak F., Maas B., Dörffling K. (2002). Importance of abscisic acid for chilling tolerance of maize seedlings. J. Plant Physiol..

[28] Vishwakarma K., Upadhyay N., Kumar N., Yadav G., Singh J., Mishra R.K., Kumar V., Verma R., Upadhyay R.G., Pandey M. (2017). Abscisic acid signaling and abiotic stress tolerance in plants: A review on current knowledge and future prospects. Front. Plant Sci..

[29] Scharwies J.D., Dinneny J.R. (2019). Water transport, perception, and response in plants. J. Plant Res..

[30] Trejo C.L., Clephan A.L., Davies W.J. (1995). How do stomata read abscisic acid signals?. Plant Physiol..

[31] Płażek A., Dubert F., Janowiak F., Krępski T., Tatrzańska M. (2011). Plant age and in vitro or in vivo propagation considerably affect cold tolerance of *Miscanthus* × *giganteus*. Eur. J. Agron..

[32] Parent B., Hachez C., Redondo E., Simonneau T., Chaumont F., Tardieu F. (2009). Drought and abscisic acid effects on aquaporin content translate into changes in hydraulic conductivity and leaf growth rate: A trans-scale approach. Plant Physiol..

[33] Finkelstein R. (2013). Abscisic acid synthesis and response. Arab. Book.

[34] Antoni R., Gonzalez-Guzman M., Rodriguez L., Peirats-Llobet M., Pizzio G.A., Fernandez M.A., De Winne N., De Jaeger G., Dietrich D., Bennett M.J. (2013). PYRABACTIN RESISTANCE_1_-LIKE8 plays an important role for the regulation of abscisic acid signaling in root. Plant Physiol..

[35] Moriwaki T., Miyazawa Y., Kobayashi A., Takahashi H. (2013). Molecular mechanisms of hydrotropism in seedling roots of *Arabidopsis thaliana* (*Brassicaceae*). Am. J. Bot..

[36] Williams J.G.K., Kubelik A.R., Livak K.J., Rafalski J.A., Tingey S.V. (1990). DNA polymorphisms amplified by arbitrary primers are useful as genetic-markers. Nucleic Acids Res..

[37] Mucciarelli M., Ferrazzini D., Belletti P. (2014). Genetic variability and population divergence in the rare *Fritillaria tubiformis* subsp. *moggridgei* Rix (Liliaceae) as revealed by RAPD analysis. PLoS ONE.

[38] Krebs C., Mahy G., Matthies D., Schaffner U., Tiébré M.-S., Bizoux J.-P. (2010). Taxa distribution and RAPD markers indicate different origin and regional differentiation of hybrids in the invasive Fallopia complex in central-western Europe. Plant Biol..

[39] Huang H., Guo S. (2005). Analysis of population genetic differences of the invasive plant (*Solidago canadensis*). Zhiwu Yanjiu.

[40] Czortek P., Królak E., Borkowska L., Bielecka A. (2020). Impacts of soil properties and functional diversity on the performance of invasive plant species *Solidago canadensis* L. on post-agricultural wastelands. Sci. Total Environ..

[41] Eckert S., Herden J., Stift M., Durka W., van Kleunen M., Joshi J. (2022). Traces of genetic but not epigenetic adaptation in the invasive goldenrod *Solidago canadensis* despite the absence of population structure. Front. Ecol. Evol..

[42] Herrera C.M., Medrano M., Bazaga P. (2016). Comparative spatial genetics and epigenetics of plant populations: Heuristic value and a proof of concept. Mol. Ecol..

[43] Eamus D., Huete A., Yu Q. (2016). Vegetation Dynamics: A Synthesis of Plant Ecophysiology, Remote Sensing and Modelling.

[44] Gupta N., Ram H., Kumar B. (2016). Mechanism of Zinc absorption in plants: Uptake, transport, translocation and accumulation. Rev. Environ. Sci. Biotechnol..

[45] Collin S., Baskar A., Geevarghese D.M., Ali M.N.V.S., Bahubali P., Choudhary R., Lvov V., Tovar G.I., Senatov F., Koppala S. (2022). Bioaccumulation of lead (Pb) and its effects in plants: A review. J. Hazard. Mater..

[46] Bączek-Kwinta R., Antonkiewicz J. (2022). Differential physiological response and potential toxicological risk of white cabbage grown in zinc-spiked soil. Agronomy.

[47] Dambiec M., Klink A., Polechońska L. (2022). Concentration and translocation of trace metals in *Solidago gigantea* in urban areas: A potential bioindicator. Int. J. Environ. Sci. Technol..

[48] Jakobs G., Weber E., Edwards P.J. (2004). Introduced plants of the invasive *Solidago gigantea* (*Asteraceae*) are larger and grow denser than conspecifics in the native range. Divers. Distrib..

[49] Popova E., Kim H.-H., Khasim S.M., Hegde S.N., González-Arnao M.T., Thammasiri K. (2020). Cryobiotechnology of Korean orchid biodiversity: A case study using *Cymbidium kanran*. Orchid Biology: Recent Trends & Challenges.

[50] Pagter M., Lefevre I., Arora R., Hausman J.-F. (2011). Quantitative and qualitative changes in carbohydrates associated with spring deacclimation in contrasting *Hydrangea* species. Environ. Exp. Bot..

[51] Borah A., Hazarika K., Khayer S.M. (2015). Drying kinetics of whole and sliced turmeric rhizomes (*Curcuma longa* L.) in a solar conduction dryer. Inf. Process. Agric..

[52] Gupta A.K., Kaur N. (2005). Sugar signalling and gene expression in relation to carbohydrate metabolism under abiotic stresses in plants. J. Biosci..

[53] Rapacz M., Tokarz K., Janowiak F. (2001). The initiation of elongation growth during long-term low-temperature stay of spring-type oilseed rape may trigger loss of frost resistance and changes in photosynthetic apparatus. Plant Sci..

[54] Xu W., Jia L., Shi W., Liang J., Zhou F., Li Q., Zhang J. (2013). Abscisic acid accumulation modulates auxin transport in the root tip to enhance proton secretion for maintaining root growth under moderate water stress. New Phytol..

[55] Dong J., Jones R.H., Mou P. (2018). Relationships between nutrient heterogeneity, root growth, and hormones: Evidence for interspecific variation. Plants.

[56] Liao Y., Zou H.-F., Wei W., Hao Y.-J., Tian A.-G., Huang J., Liu Y.-F., Zhang J.-S., Chen S.-Y. (2008). Soybean GmbZIP44, GmbZIP62 and GmbZIP78 genes function as negative regulator of ABA signaling and confer salt and freezing tolerance in transgenic *Arabidopsis*. Planta.

[57] Pagter M., Jensen C.R., Petersen K.K., Liu F., Arora R. (2008). Changes in carbohydrates, ABA and bark proteins during seasonal cold acclimation and deacclimation in Hydrangea species differing in cold hardiness. Physiol. Plant..

[58] Ortega-Martinez O., Pernas M., Carol R.J., Dolan L. (2007). Ethylene modulates stem cell division in the *Arabidopsis thaliana* root. Science.

[59] Sarkar A.K., Luijten M., Miyashima S., Lenhard M., Hashimoto T., Nakajima K., Scheres B., Heidstra R., Laux T. (2007). Conserved factors regulate signalling in *Arabidopsis thaliana* shoot and root stem cell organizers. Nature.

[60] Charpentier M., Sun J.H., Wen J.Q., Mysore K.S., Oldroyd G.E.D. (2014). Abscisic acid promotion of arbuscular mycorrhizal colonization requires a component of the PROTEIN PHOSPHATASE 2A Complex(1 W OPEN). Plant Physiol..

[61] Herrera-Medina M.J., Steinkellner S., Vierheilig H., Bote J.A.O., Garrido J.M.G. (2007). Abscisic acid determines arbuscule development and functionality in the tomato arbuscular mycorrhiza. New Phytol..

[62] Abrahamson W.G., Gadgil M. (1973). Growth form and reproductive effort in goldenrods (Solidago, Compositae). Am. Nat..

[63] Conti L., Galbiati M., Tonelli C., Zhang D.-P. (2014). ABA and the floralt transition. Abscisic Acid: Metabolism, Transport and Signaling.

[64] Nambara E., Okamoto M., Tatematsu K., Yano R., Seo M., Kamiya Y. (2010). Abscisic acid and the control of seed dormancy and germination. Seed Sci. Res..

[65] Brookbank B.P., Patel J., Gazzarrini S., Nambara E. (2021). Role of basal ABA in plant growth and development. Genes.

[66] Zhao H., Peng S., Chen Z., Wu Z., Zhou G., Wang X., Qiu Z. (2011). Abscisic Acid in Soil Facilitates Community Succession in Three Forests in China. J. Chem. Ecol..

[67] Zhang Y., Xu F., Ding Y., Du H., Zhang Q., Dang X., Cao Y., Dodd I.C., Xu W. (2021). Abscisic acid mediates barley rhizosheath formation under mild soil drying by promoting root hair growth and auxin response. Plant Cell Environ..

[68] Basirat M., Mousavi S.M., Abbaszadeh S., Ebrahimi M., Zarebanadkouki M. (2019). The rhizosheath: A potential root trait helping plants to tolerate drought stress. Plant Soil.

[69] Aslam M.M., Karanja J.K., Dodd I.C., Waseem M., Weifeng X. (2022). Rhizosheath: An adaptive root trait to improve plant tolerance to phosphorus and water deficits?. Plant Cell Environ..

[70] Pellicone G., Caloiero T., Guagliardi I. (2019). The De Martonne aridity index in Calabria (Southern Italy). J. Maps.

[71] Ellenberg H. (1988). Vegetation Ecology of Central Europe.

[72] Stojanovic D.B., Krzic A., Matovic B., Orlovic S., Duputie A., Djurdjevic V., Galic Z., Stojnic S. (2013). Prediction of the European beech (*Fagus sylvatica* L.) xeric limit using a regional climate model: An example from southeast Europe. Agric. For. Meteorol..

[73] Jarosińska E., Bodziony M. (2019). Czasowo-przestrzenna zmienność deszczu na zurbanizowanym obszarze Krakowa. Acta Sci. Pol. Form. Circumiectus.

[74] Cassol D., De Silva F.S.P., Falqueto A.R., Bacarin M.A. (2008). An evaluation of non-destructive methods to estimate total chlorophyll content. Photosynthetica.

[75] Bączek-Kwinta R., Juzoń K., Borek M., Antonkiewicz J. (2019). Photosynthetic response of cabbage in cadmium-spiked soil. Photosynthetica.

[76] Borek M., Bączek-Kwinta R., Rapacz M. (2016). Photosynthetic activity of variegated leaves of *Coleus x hybridus* hort. cultivars characterised by chlorophyll fluorescence techniques. Photosynthetica.

[77] Jones J.B., Case V.W., Westerman R.L. (1990). Sampling, handling, and analyzing plant tissue samples. Soil Testing and Plant Analysis.

[78] Ostrowska A., Gawliński S., Szczubiałka Z. (1991). Methods of Analysis and Assessment of Soil and Plant Properties.

[79] van de Wiel H.J. (2003). Determination of Elements by ICP-AES and ICP-MS.

[80] Wedepohl K.H. (1995). The composition of the continental-crust. Geochim. Cosmochim. Acta.

[81] Gope M., Masto R.E., George J., Hoque R.R., Balachandran S. (2017). Bioavailability and health risk of some potentially toxic elements (Cd, Cu, Pb and Zn) in street dust of Asansol, India. Ecotoxicol. Environ. Saf..

[82] Kabir M.H., Rashid M.H., Wang Q.Y., Wang W.Q., Lu S.L., Yonemochi S. (2021). Determination of Heavy Metal Contamination and Pollution Indices of Roadside Dust in Dhaka City, Bangladesh. Processes.

[83] Simlat M., Ptak A., Kula A., Orzel A. (2018). Assessment of genetic variability among raspberry accessions using molecular markers. Acta Sci. Pol.-Hortorum Cultus.

[84] Ashwell G. (1957). Colorimetric analysis of sugars. Methods Enzymol..

[85] Bates L.S., Waldren R.P., Teare I.D. (1973). Rapid determination of free proline for water-stress studies. Plant Soil.

[86] Carillo P., Mastrolonardo G., Nacca F., Parisi D., Verlotta A., Fuggi A. (2008). Nitrogen metabolism in durum wheat under salinity: Accumulation of proline and glycine betaine. Funct. Plant Biol..

[87] Hummel I., Pantin F., Sulpice R., Piques M., Rolland G., Dauzat M., Christophe A., Pervent M., Bouteille M., Stitt M. (2010). Arabidopsis plants acclimate to water deficit at low cost through changes of carbon usage: An integrated perspective using growth, metabolite, enzyme, and gene expression analysis. Plant Physiol..

[88] Walker-Simmons M.K., Abrams S.R., Davies W.J., Jones H.G. (1991). Use of ABA immunoassays. Abscisic Acid, Physiology and Biochemistry.

